# Context and meter enhance long-range planning in music performance

**DOI:** 10.3389/fnhum.2014.01040

**Published:** 2015-01-13

**Authors:** Brian Mathias, Peter Q. Pfordresher, Caroline Palmer

**Affiliations:** ^1^Department of Psychology, McGill UniversityMontreal, QC, Canada; ^2^Department of Psychology, University at Buffalo, State University of New YorkBuffalo, NY, USA

**Keywords:** memory retrieval, sequence learning, music performance, meter, planning

## Abstract

Neural responses demonstrate evidence of resonance, or oscillation, during the production of periodic auditory events. Music contains periodic auditory events that give rise to a sense of beat, which in turn generates a sense of meter on the basis of multiple periodicities. Metrical hierarchies may aid memory for music by facilitating similarity-based associations among sequence events at different periodic distances that unfold in longer contexts. A fundamental question is how metrical associations arising from a musical context influence memory during music performance. Longer contexts may facilitate metrical associations at higher hierarchical levels more than shorter contexts, a prediction of the range model, a formal model of planning processes in music performance (Palmer and Pfordresher, 2003; Pfordresher et al., 2007). Serial ordering errors, in which intended sequence events are produced in incorrect sequence positions, were measured as skilled pianists performed musical pieces that contained excerpts embedded in long or short musical contexts. Pitch errors arose from metrically similar positions and further sequential distances more often when the excerpt was embedded in long contexts compared to short contexts. Musicians’ keystroke intensities and error rates also revealed influences of metrical hierarchies, which differed for performances in long and short contexts. The range model accounted for contextual effects and provided better fits to empirical findings when metrical associations between sequence events were included. Longer sequence contexts may facilitate planning during sequence production by increasing conceptual similarity between hierarchically associated events. These findings are consistent with the notion that neural oscillations at multiple periodicities may strengthen metrical associations across sequence events during planning.

## Introduction

Whether dialing a telephone number, playing a musical instrument, or carrying out a surgical operation, people learn and remember the serial order of the actions they produce. Early theoretical accounts of sequence production focused on the role of simple event associations, which triggered the successive actions to be produced in a sequence (Watson, 1920; Wickelgren, 1965). Sequences are often produced too quickly, however, for individuals to rely on feedback for production of upcoming events (Lashley, 1951). The presence of repeated sequence elements, such as musical pitches, also requires a mechanism by which multiple paired associations for the same item could be distinguished, which association theories lack (Baddeley, 1968; Henson et al., 1996). It is therefore more likely that performance is guided by a mental plan: internal representations that specify which and when different sequence events must be performed (Miller et al., 1960; Schank and Abelson, 1977).

Most modern theories of serial order in sequence production propose that mental planning or preparation of events extends incrementally during performance to a subset of sequence events (Rumelhart and Norman, 1982; Dell et al., 1997; Palmer and Pfordresher, 2003; Pfordresher et al., 2007). In incremental frameworks, producers’ access to subsets or increments of sequence events evolves over the course of production, and incremental planning co-occurs with execution. Retrieval of events from memory in the correct serial order is achieved by stepping in cascading fashion through a series of activation-based incremental representations (Houghton, 1990; Burgess and Hitch, 1992; Palmer and Pfordresher, 2003; Pfordresher et al., 2007). The success of incremental models is noted in speech and music (Dell et al., 1997; Palmer and Pfordresher, 2003), in which complex hierarchical relationships exist among sequence events. Incremental models for planning of speech and music are contextual; the contents of an incremental plan are defined for events relative to the context in which they occur.

Although contextual relationships can aid memory for sequence events, the context can also contribute to forgetting. As the number of elements in a list context increases, the accuracy with which the list is serially recalled decreases, when list contents are ordered randomly (Miller, 1956; Crannell and Parrish, 1957) and less so when they are structured, as in music (Finney and Palmer, 2003). List length effects on forgetting have been attributed to the decay of list information with the passage of time (Bjork and Whitten, 1974; Crowder, 1976; Neath, 1993; Brown et al., 2000), interference of later list items with previous list items that are held in working memory (Nairne, 1990; Henson, 1998; Farrell and Lewandowsky, 2004), or displacement of previous list items by new ones (Waugh and Norman, 1965; Cowan, 2001). Although hierarchical similarity relationships among sequence events can reduce list length effects, longer structured lists are generally more difficult to remember than shorter lists.

**Figure 1:**
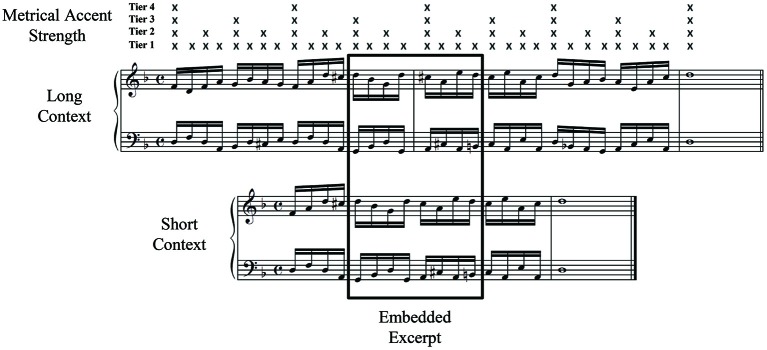
**Sample musical excerpt (in rectangle) with its associated long and short surrounding contexts**. Metrical accent strengths according to a 4-tier metrical hierarchy are depicted by Xs. Events aligned with higher tiers receive stronger accent.

Contextual relationships among sequence elements are often described in terms of hierarchies, or multi-leveled associations. Hierarchical relationships may speed short-term recall processes by reducing working memory demands (Houghton and Hartley, 1995; Schneider and Logan, 2007). Music performance is a classic example of serial ordering behavior and provides a fertile testing ground for investigations of sequential planning (Palmer, 2005). Musical meter, in particular, may influence planning during production. Musical meter refers to a perceived alternation of strong and weak accents that recur at different periodicities in musical sequences to form a metrical hierarchy (Lerdahl and Jackendoff, 1983). Figure 1 shows the accent strength of each event in a musical sequence, predicted from the event’s placement in a metrical grid. The accent strength of each event is indicated by the Xs above the music notation. Musical events that possess the same metrical accent strength (i.e., belong to the same metrical hierarchical level) are perceived as more similar (Palmer and Krumhansl, 1990; Palmer and van de Sande, 1993). Performers tend to mark events aligned with varying degrees of metrical accent strength through variations in timing, intensity, and articulation (Drake and Palmer, 1993), and they are also more accurate in producing duration patterns that match a metrical framework than those that do not match as well (Povel, 1981; Essens and Povel, 1985). Thus, several sources of evidence suggest that meter serves as a hierarchical framework that aids the planning and execution of musical events in performance (Palmer and Pfordresher, 2003; Pfordresher et al., 2007).

Serial ordering errors, in which intended sequence events are produced in an incorrect order, serve as evidence that multiple events are simultaneously accessible in memory during production. Serial ordering errors have been studied extensively in speech (cf. Boomer and Laver, 1968; MacKay, 1970; Garrett, 1980; Dell, 1986; Bock, 1995) and in music (cf. Palmer and van de Sande, 1993, 1995; Palmer and Drake, 1997; Drake and Palmer, 2000; Palmer and Pfordresher, 2003; Pfordresher et al., 2007). Errors in both domains tend to reflect interactions among intended sequence events that are similar on structural dimensions (MacKay, 1970; Garrett, 1975; Dell and Reich, 1981). In music performance, for example, serial ordering errors occur often between events that have similar amounts of metrical accent (Palmer and Pfordresher, 2003). Serial ordering errors also tend to arise from proximal sequence distances; the probability of an item’s recall in a particular serial position tends to decrease with distance from the correct position (MacKay, 1970; Healy, 1974; Nairne, 1990). The distance between an item’s correct (intended) position and its incorrect position in music performance errors suggests that memory for an item is active over 3–4 sequence positions on average, with roughly equivalent proportions of anticipatory and perseveratory errors (Pfordresher et al., 2007). This span of sequence positions is referred to as a performer’s range of planning (Palmer and van de Sande, 1995; Palmer and Pfordresher, 2003). Proximity effects on serial recall are attributed to both decay and interference (MacKay, 1970; Palmer and van de Sande, 1993, 1995; Palmer and Pfordresher, 2003; Noteboom, 2005; Pfordresher et al., 2007).

Both structural similarity and serial decay processes in memory have been described in terms of neural mechanisms. Periodic oscillations of neural firing have been modeled with nonlinear dynamical systems to capture listeners’ attentional entrainment to quasi-periodic metrical structure seen in music (Large and Kolen, 1994; Large and Palmer, 2002). Different neural oscillators can operate at different metrical periods representing hierarchical levels, and may track or predict upcoming musical beats (Large and Jones, 1999). Serial decay of memory has been formulated in terms of a reduction in the activation that spreads from node to node within a neural network (Dell, 1986; Acheson et al., 2011). To-be-recalled memories are thought be stored diffusely throughout cortex and function by association (Fuster, 1997). The range model of memory retrieval (Palmer and Pfordresher, 2003; Pfordresher et al., 2007) quantifies potential mechanisms related to metrical similarity and serial decay in item selection and retrieval, as well as performance accuracy, and predicts both rates and types of serial ordering errors during music performance. The model predicts a greater role of metrical similarity in the planning of sequence events that are embedded in longer contexts than in shorter contexts, due to increased activation of more events aligned with higher (more distant) hierarchical levels. This prediction contrasts with theories proposing greater interference for events placed in longer contexts than in shorter contexts, in concurrence with list length effects. In the following section, we review the range model and its assumptions. We then describe a study that tests these assumptions using measurements of pianists’ serial ordering errors, keystroke intensities, and error rates in a speeded music performance task.

### The range model of planning

The range model (Palmer and Pfordresher, 2003; Pfordresher et al., 2007) is a contextual planning model that describes how musical events are retrieved from memory during performance. It predicts the accuracy of performance, as well as types of errors that performers occasionally produce, based on planning processes. The range model differs from other contextual models of memory retrieval in two key ways. First, the model predicts increased event activation according to similarity-based measures of metrical accent. Second, the range model specifies the relationship between the amount of context in a sequence and the accessibility of the current event based on a parameter that reflects constraints on short-term memory.

Contextual event activations in the range model (Palmer and Pfordresher, 2003) are computed with the multiplication of two distinct model components: a serial proximity component (*S*) and a metrical similarity component (*M*). One range model component contributing to the strength of contextual event activations is the serial proximity component (*S*). The serial proximity component predicts a graded decrease in activation of contextual events as the distance between the current event and the contextual events increases. The most active contextual events are located at positions closest to the current event and the least active at positions furthest away. Contextual activations (*S_x_*) are modeled by the equation

(1) $$S_{x}=a^{\left(\left|x\right|/t\right)}$$

where *a* is a free parameter that ranges from 0.8 < *a* ≤ 1.0, representing constraints on working memory that can change with development and task-specific memory demands. The distance (*x*) is the number of events between the current event and another sequence event; the contextual activations are weighted by the serial distance between them. The *t* parameter, limited to durations typical of musical sequences, 0.1 < *t* ≤ 2.0 s, represents the duration of a single event and is determined by production rate (interonset interval (IOI), in *s*). As the parameters *t* or *a* decrease, the serial component predicts a faster drop-off in activation strength with sequence distance. An important implication is that events surrounding the current event are less active when events are produced at faster rates (smaller values of *t*) or with higher constraints on working memory (smaller values of *a*). The model assumes that event activations increase as tempo slows (larger *t*) because more time is available for performers to retrieve information about the surrounding context while preparing events for production, as supported by the fact that errors tend to arise from nearby contextual positions at faster tempi.

Another range model component contributing to the strength of contextual event activations is the metrical similarity component (*M*). Metrical similarity between the current event and each contextual event at distance *x* within one metrical cycle of position *i* is computed as

(2) $$M_{x}\left(i\right)=1-\frac{\left|m_{i}-m_{i+x}\right|}{m_{i}+m_{i+x}}$$

where *M* is a vector of similarity relations defined between the current event and all sequence elements (see Palmer and Pfordresher, 2003, for further information). Metrical accent strengths *m_i_*, represented by metrical grids proposed for Western tonal music (Cooper and Meyer, 1960; Lerdahl and Jackendoff, 1983), are indicated by Xs in Figure 1. The more metrical levels (*j*) with which an event at position *i* is associated, the stronger the accent. The ratio in Equation 2 reflects a generalized form of Weber’s law; perceptual sensitivity to a difference between any two metrical accents depends on the absolute difference between the two events’ metrical levels. The metrical similarity computations, when averaged across current event positions, predict an alternation of high and low metrical similarity across sequence positions, which matches error proportions measured in pianists’ serial ordering errors (Palmer and Pfordresher, 2003).

There is often one metrical level that is more salient than other metrical levels; this level is referred to as the tactus, and is usually the level at which people clap or tap to the beat (Lerdahl and Jackendoff, 1983). The range model’s metrical component introduces a second free parameter, *w_j_*, that is applied to the tactus and enhances the strength of accents at that particular level. The metrical weighting of all levels is computed as a proportion that sums to 1 across levels. Therefore, if 4 levels were weighted equally, *w_j_* would take the value 0.25. If a performance is judged to have a tactus, as is usually the case, *w_j_* for that level is assumed to exceed 0.25. If the tactus were metrical level 2 (*j* = 2), for example, the weights applied to the levels 1, 3, and 4 would equal ((1−*w*_2_)/(*k*−1)), where *k* represents the total number of metrical levels, to ensure that weights across all levels sum to 1. The metrical accent strength of each event in a musical sequence, *m_i_*, is therefore determined by multiplying the sum of the metrical accents present at each position *i* and metrical level* j*, *g_ji_*, with the weight, *w_j_*, at each level:

(3) $$m_{i}=\sum_{j=1}^{k}w_{j}*g_{ji}$$

The values of *m_i_* are shown in Figure 1 above a sample musical piece from the current study, which were structured with 4 metrical levels in which the lowest level was the sixteenth-note level, similar to previous studies (Palmer and Pfordresher, 2003; Pfordresher et al., 2007).

Absolute activations of sequence events are computed by multiplying the range model’s serial component (*S*) by the metrical component (*M*). The nonlinear (multiplicative) relationship between serial and metrical components, shown in Equation 4, describes serial ordering error patterns in music performance better than other models, including an additive model (for further model comparisons see Palmer and Pfordresher, 2003). Absolute activation values are assumed to be updated in working memory for each event as the sequence is produced.

Equations 1 to 3 predict the conditional probability that errors arise from a given source, assuming that an error has occurred. In order to model the probability of making an error, the absolute activation of sequence events was defined in an extended version of the range model (Pfordresher et al., 2007), which proposed that the activation of the current event grows in proportion to the squared sum of the contextual events’ activations:

(4) $$Event_{\left(0\right)}=S_{0}*M_{0}={\left(\sum_{x\neq 0}S_{x}*M_{x}\right)}^{2}$$

This equation is based on the model’s theoretical assumption that performers use information about the surrounding context while retrieving the current event; the activation of the current event is defined by the activations of surrounding events. *Event*_(0)_ refers to the current event, for which distance *x* = 0. Contextual events (all events other than the current event) are specified by *x* ≠ 0. On average, the model predicts equivalent activation for contextual events before and after the current event, due to the symmetrical relationship of the serial component.

**Figure 2:**
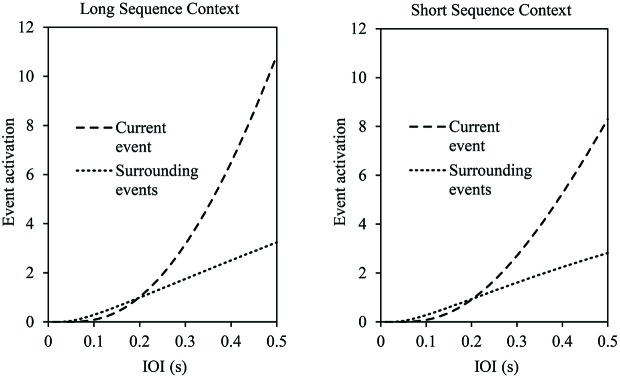
**Range model predictions of absolute activation of the current event and surrounding events by production rate (interonset interval (IOI), in s) in a long sequence context (left panel: 33 events) and short sequence context (right panel: 17 events)**. Current and surrounding event activations are based on averages across all excerpt positions. Model parameters fixed for these predictions: *a* = 0.85, *B* = 1, *w_j_* = 0.25.

The range model also makes predictions related to the length of a sequence context that we test here. Figure 2 shows the model’s predictions for the activation of the current event and its surrounding events within a long context (left panel) and within a short context (right panel) as a function of production rate. As shown, the current event’s activation increases exponentially as the production rate slows down (increased IOI), and the increased activation is steeper for long contexts than for short contexts. Thus, longer contexts, which contain more metrically similar events than shorter contexts, facilitate retrieval of the current event.

### Range model predictions

The assumptions of the range model yield several predictions regarding the relative importance of highly accented metrical events in longer musical contexts. First, longer sequence contexts yield greater memory activation for the event currently being produced and its contextual sequence events, resulting in a larger range of planning for longer contexts. This prediction is shown in Figure 2; as the sequence context grows, the activation of the current event exceeds that of other context events. Second, increased numbers of highly accented metrical events in longer contexts lead to the prediction of greater metrical similarity overall, as a function of the Weber’s law relationship in Equation 2. Third, increased numbers of highly accented metrical events should reduce error rates for events placed in longer contexts compared to shorter contexts, due to increased metrical similarity.

We tested these predictions by manipulating the size of sequence context available to performers as they produced musical sequences at fast and slow production rates. Pianists’ serial ordering errors and tone intensities (corresponding to loudness) were compared for musical excerpts that were placed in long or short sequence contexts. We tested the prediction that the mean range (distance between an error and its intended serial position) was greater when excerpts were surrounded by long contexts compared to short contexts. We also tested the prediction that longer contexts should enhance hierarchical relationships between events with similar metrical accent strength; therefore, we expected greater metrical similarity for serial ordering errors that occurred in excerpts placed in long contexts compared to short contexts. Finally, we tested the prediction that pitch error rates would show stronger effects of metrical hierarchies when the excerpts were surrounded by long contexts compared to short contexts. We fit the range model to mean and individual subject data, and tested whether the model’s metrical component accounted for increased influence of context on performance errors.

## Materials and methods

### Participants

Twenty-six experienced adult pianists from the Montreal music community (*M* age = 22.9 years, range = 18–31, *SD* = 7.7) participated. Participants had a mean of 15.8 years of experience performing the piano (range = 7–29 years, *SD* = 7.6). All participants reported playing the instrument regularly and none reported having any hearing problems. Of the 26 participants, 24 reported being right-handed and 2 were ambidextrous; 16 were female. Participants provided written informed consent, and the study was conducted in accordance with the principles laid down in the Declaration of Helsinki and approved by the McGill University Research Ethics Board.

### Materials

Eight musical excerpts conforming to conventions of classical Western tonal music were composed for the experiment. Each excerpt contained a total of 16 isochronous sixteenth notes (8 for the right and 8 for the left hand) in 4/4 meter. Each musical excerpt was then embedded in one long context (12 sixteenth-note events preceding and 13 following) and one short context (4 sixteenth-note events preceding and 5 following), which were composed of isochronous sixteenth notes for each hand, followed by a final whole note; thus, all sequence locations contained equivalent numbers of events across excerpts, in order to examine effects of context at all metrical positions. Figure 1 shows one of the excerpts (boxed) placed in a long context (top) and a short context (bottom). Embedding the same musical excerpt in long and short contexts permitted experimental control of the frequency of pitch reoccurrence, progressions of harmony, changes in hand position, and musical key. The 4 events immediately surrounding each excerpt were also identical across long and short contexts. Half of the pieces were in major keys (two in C Major, and one each in G Major and F Major) and the other half were in minor keys, based on harmonic minor scales (C minor, G minor, D minor, and A minor). To ensure that pianists used the same finger movements for performances of the same musical excerpt, finger numbers were provided within the music notation above or below pitches where there was ambiguity regarding finger choice (at the beginnings of the sequences and at certain pitch change points). Similar to phonemic repetitions in tongue-twisters (Garrett, 1980), the musical pieces established pitch contour and repetition patterns early in the stimulus that were violated later in the stimulus. Pitches repeated every 6 events on average and no pitches repeated successively.

### Apparatus

The sequences were performed by pianists on a Roland RD-700 electronic keyboard. All auditory feedback was delivered to participants through AKG K271 Studio headphones, with an Edirol Studio Canvas SD-80. A Classical A02 Piano1d Sound Canvas timbre was used for piano tones and a classical drum timbre was used for metronomic clicks at the beginnings of trials. FTAP computer software controlled the timing of metronome ticks and recorded the pitch, timing, and MIDI velocity of all key press events (Finney, 2001).

### Design and procedure

In a within-subjects design, participants were presented with 2 contexts (long/short) × 2 tempi (fast/medium) × 4 test blocks (1–4). To test the model’s predictions, each pianist performed half of the excerpts placed in long contexts and the other half placed in short contexts, and each pianist performed half of the pieces at a medium tempo (225 ms per sixteenth-note IOI, or 67 beats per minute) and the other half at a fast tempo (187.5 ms per sixteenth-note IOI, or 80 beats per minute). The pieces assigned to each tempo condition were counterbalanced across participants. The experiment consisted of a learning phase, in which participants learned all of the musical stimuli, and a test phase, in which each piece was performed 2 times per block over 4 test blocks for a total of 8 test performances. In the test phase, long and short contexts were grouped within each block and half of the pianists performed excerpts surrounded by long contexts first, and the other half performed excerpts surrounded by short contexts first. Presentation order of long and short contexts was manipulated in a Latin square design across blocks. Medium and fast production rates alternated every trial and their order was counterbalanced across participants. Thus, the test phase of the experiment contained a total of 4 (excerpts) × 2 (tempi) × 4 (blocks) × 2 (repetitions) = 64 trials.

At the start of the session, participants completed a questionnaire about their musical background. Participants then completed the learning phase of the experiment. During the learning phase, participants were presented with the music notation for one of the musical stimuli (a musical excerpt embedded in a context) and were asked to practice the piece, for a maximum of 3 min, until they felt comfortable playing it at a slow tempo. Participants were then asked to perform the piece using the notated fingering at a slow tempo of 429 ms per sixteenth-note IOI (35 beats per minute), as indicated by an initial metronome beat that sounded on the quarter note. Participants were given three attempts to perform each piece without making any errors at this slow tempo before moving on to the next piece. Piece orders were counterbalanced; the same order was used for the learning phase and the first block of the test phase of the experiment. All participants were able to perform every piece without errors at the slow tempo before continuing to the test phase of the experiment. Thus, all pianists learned the 8 excerpts in their associated contexts to a note-perfect criterion (pianists were never presented with the excerpts in isolation). These slow performances ensured that errors that occurred at faster tempi were not due to sight-reading failures or incorrect learning of the musical pieces.

Following the learning phase of the experiment, participants were asked to perform the pieces at the faster tempi in a test phase consisting of 4 experimental blocks. In each trial, participants first heard a metronome click to establish the prescribed tempo, which sounded at the quarter-note level throughout each trial. Participants were instructed to perform the sequence at the tempo indicated by the metronome using the notated fingering and, if they made any errors, to continue playing and not stop or try to correct them. They performed the piece twice in each trial, pausing between repetitions, with the musical notation in view. The entire experiment lasted 60 min, and participants received a nominal fee for their participation.

### Data analysis

Pitch errors were identified by computer comparison of pianists’ performances with the information in the notated musical score (Large, 1993; Palmer and van de Sande, 1993, 1995). The majority of errors involved an intruder (unintended event) that replaced a target (intended event, as determined by the musical notation). The assumption that notated events were intended is based on the participants’ error-free performances at the slower tempo. The intruder could reflect a pitch that was coded as contextual (present in the musical stimulus) or noncontextual (a pitch not found anywhere in the musical stimulus). Contextual errors, also called movement errors (Dell et al., 1997; Palmer and Pfordresher, 2003), were coded for distance in terms of the number of intervening events (sixteenth notes) between the error and its source (nearest same pitch) in the musical score. An exchange of two successive pitches in serial order (for example, performing A-D-E-C when the intended sequence was A-E-D-C) was coded as a single contextual error (exchange), to be consistent with previous coding systems. The coding of pitch errors was octave-specific, and errors were coded in terms of the nearest pitch in both frequency (pitch height) and time. Multiple incorrect pitches with onsets occurring within the same small temporal window (94 ms, approximately half of the fastest tempo, which was 187.5 ms IOI) were coded as a single error (a chord error). Noncontextual errors consisted of deletions and additions (additions of pitches that were not intended for anywhere in the entire piece). Analyses of error intensities and error rates were conducted on all errors (contextual and noncontextual) produced within the stimulus excerpts. To avoid errors that arose from sight-reading tasks or learning problems, any pitch error that occurred in one of the 3 initial slow performances during the learning phase and in more than half (4 or more performances) of the total 8 test phase performances for the same stimulus were excluded from analyses (*n* = 21, less than 1% of all errors produced during the test phase). Although subjects were instructed not to stop to correct errors, subjects occasionally corrected errors; corrected errors were also excluded from all analyses.

The relative frequency with which contextual errors arose across sequence distances was examined in terms of a movement gradient, a distribution of error frequencies as a function of the distance of the nearest intended event from the current event (Brown et al., 2000, 2007; Vousden et al., 2000). To measure whether a context-dependent pattern of results would occur by chance, we report error simulations based on random sampling from the set of sequence pitches. The *a-priori* global alpha level was 0.05. Greenhouse-Geisser corrections were performed for analyses when necessary. Pianists’ MIDI key velocities (associated with physical intensity and subsequent perceived loudness) were analyzed in both correct and incorrect keystrokes for evidence of metrical hierarchies.

## Results

Participants performed a total of 7,469 errors across the entire musical sequences. The mean error rate per trial, which was based on all pitch errors that occurred within participants’ performances, was 0.10. Eighty percent of produced errors were contextual errors (deletion errors were the most frequently produced noncontextual error). The mean production rate across performances was 194.2 ms per tone (*SD* = 6.0 ms) in the fast condition (prescribed rate = 187.5 ms) and 226.7 ms per tone (*SD* = 3.8 ms) in the medium condition (prescribed rate = 225 ms). Thus, the participants performed at tempi close to those prescribed.

Within the excerpts, a total of 2,736 errors occurred and the mean error rate per trial was 0.12. The error rate per trial for excerpts performed within long contexts was 0.13, and the error rate for excerpts performed within short contexts was 0.11. Eighty-one percent of errors produced within excerpts were contextual errors. Eighty-three percent of errors produced within excerpts surrounded by long contexts were contextual, and 79% of errors produced within excerpts surrounded by short contexts were contextual. Ninety-three percent of all contextual errors had an identifiable source within an absolute distance of 8 events (one complete metrical cycle consisting of 8 sixteenth-note events). Thus, the types of errors and error rate produced by pianists within the excerpt were representative of those produced across the entire sequence. All further analyses are reported on the performances of the embedded musical excerpts.

### Metrical hierarchies

#### Correct tone intensities

**Figure 3:**
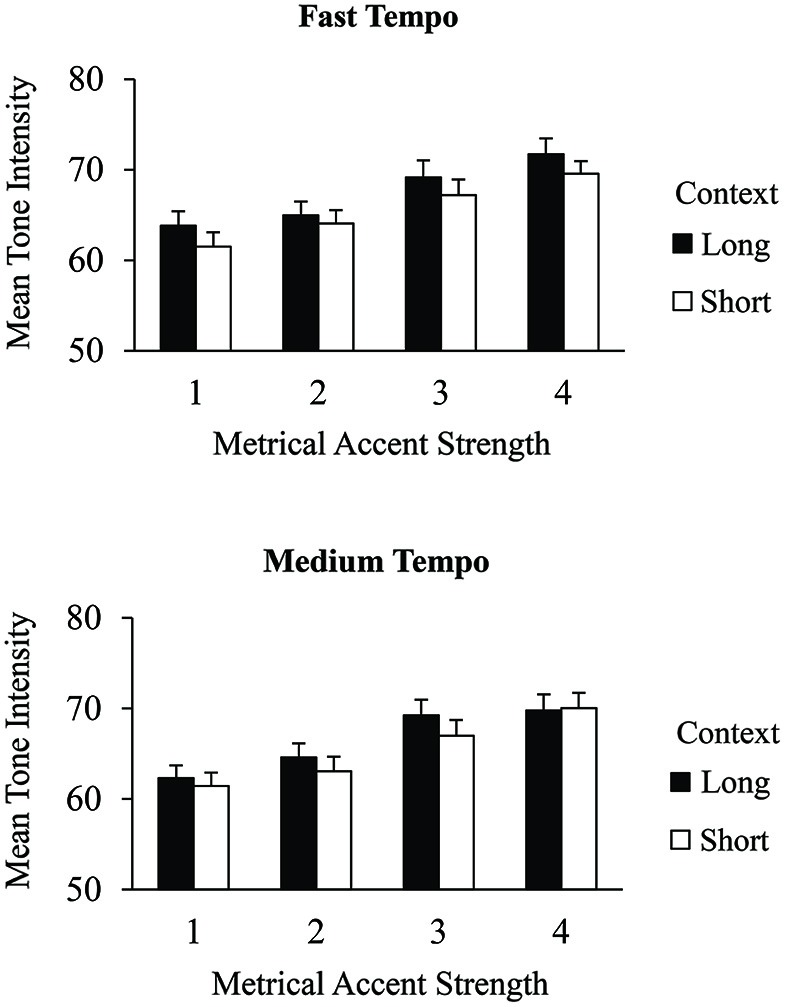
**Mean tone intensities (MIDI values) by context condition and metrical accent strength for correctly produced excerpt events performed at the fast tempo (top) and medium tempo (bottom)**. Error bars represent one standard error.

We tested the hypothesis that longer contexts would increase the salience of metrical levels by evaluating tone intensities in terms of their metrical accent strength. Figure 3 shows the mean MIDI tone intensities of correct excerpt tones that received different metrical accent strengths. A 2 (context) × 2 (tempo) × 4 (metrical accent) ANOVA indicated a main effect of metrical accent on the intensity with which tones were produced within the excerpts, *F*_(3,75)_ = 68.66, *p* < 0.001. Tones produced at more strongly accented positions were performed with greater intensity than tones produced at more weakly accented positions (Tukey HSD = 1.62, *α* = 0.05), indicating that the metrical accent manipulation reliably altered participants’ keystroke intensities. In addition, there was a main effect of context, *F*_(1,25)_ = 8.84, *p* < 0.01. Tones within long contexts were produced with greater intensity (*M* = 67.0, *SE* = 0.62) than tones within short contexts (*M* = 65.5, *SE* = 0.60). The three-way interaction between metrical accent, context, and tempo was also significant, *F*_(3,75)_ = 3.20, *p* < 0.05. *Post hoc* comparisons indicated that tones in the long contexts were produced with greater intensity when they aligned with strong metrical accent strengths (3 or 4) than with weak accent strengths (1 or 2) in long contexts, for both tempo conditions (Tukey HSD = 1.97, *α* = 0.05). Tones in short contexts also showed a significant change in intensity between strong and weak metrical accents; this difference was reduced at the medium tempo. There was no main effect of tempo on tone intensities, and there were no other significant interactions. Thus, both the metrical accent strength of individual tones and the size of the surrounding context increased the intensities with which tones were produced.

#### Error intensities

**Figure 4:**
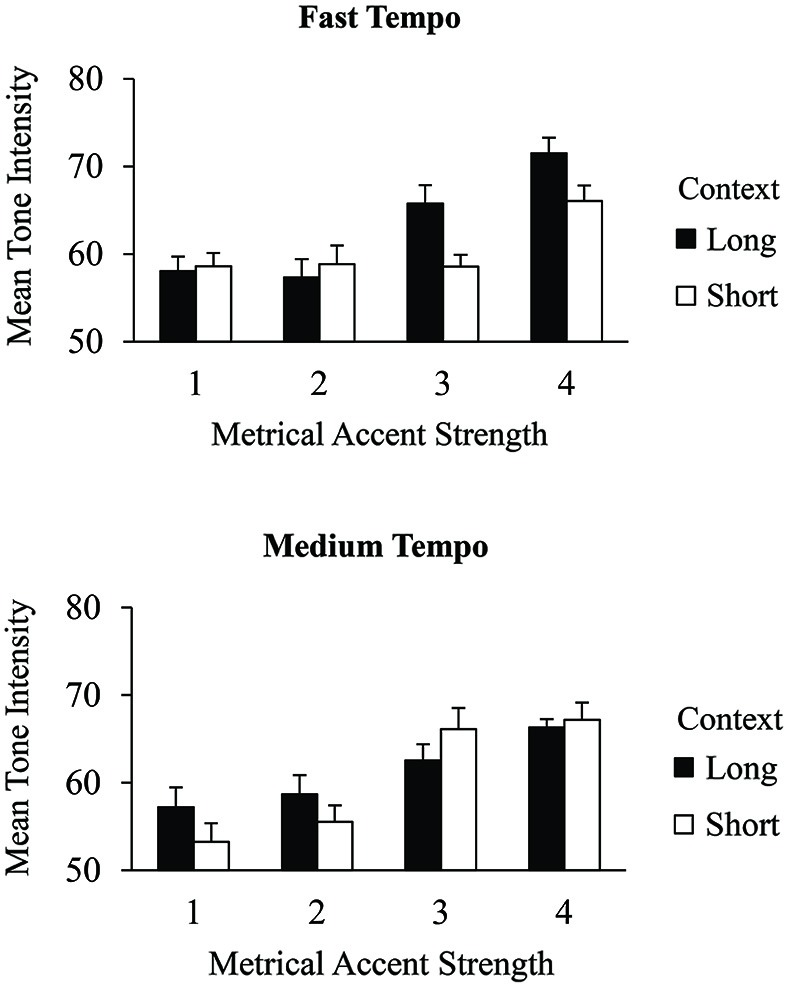
**Mean tone intensities (MIDI values) by context condition and metrical accent strength for all excerpt errors performed at the fast tempo (top) and medium tempo (bottom)**. Error bars represent one standard error.

Context effects on the salience of metrical levels were also investigated in the tone intensities of excerpt errors. Figure 4 shows the mean intensities of error events produced within the excerpts by context and metrical accent strength. A 2 (context) × 2 (tempo) × 4 (metrical accent) ANOVA indicated a main effect of metrical accent, *F*_(3,75)_ = 31.53, *p* < 0.001. As with correct events, errors produced at the most strongly accented positions were performed with greatest intensity (*M* = 67.8, *SE* = 0.84), followed by errors at the next strongest accent positions (*M* = 63.2, *SE* = 1.01), followed by the two most weakly accented positions (accent strength 2 *M* = 57.6, *SE* = 1.03; accent strength 1 *M* = 56.8, *SE* = 0.97), which did not differ from each other (Tukey HSD = 3.92, *α* = 0.05). There was a significant three-way interaction between metrical accent, context, and tempo, *F*_(3,75)_ = 8.34, *p* < 0.001. *Post hoc* comparisons indicated that, in the long context condition, tones receiving the strongest metrical accent strength (4) were performed with greater intensity than other tones (accent strengths 1, 2, and 3) at the medium tempo, but not at the fast tempo (Tukey HSD = 6.69, *α* = 0.05). The opposite pattern was found in the short context condition: Tones receiving the strongest accent strength (4) were performed with greater intensity than other tones (accent strengths 1, 2, and 3) at the fast tempo but not at the medium tempo. There were no other significant main effects or interactions. An analysis of only contextual errors in which metrical accent strength was coded in terms of the intruder position (as opposed to the target position) revealed that error intensities at metrical levels 3 and 4 did not significantly differ from those at levels 1 and 2 (Tukey HSD = 2.79, *α* = 0.05), suggesting that contextual errors adopt the intensity of the position at which they are produced. Similar to the correct events, performers produced errors with greater intensity when they were aligned with positions of strong metrical accent, and these effects were mediated by the context and tempo. Planned metrical associations may therefore differ from executed metrical accents.

### Context effects

#### Error rates

We predicted that longer contexts would enhance effects of meter on performers’ error rates. Specifically, lower error rates were expected at excerpt locations aligned with stronger metrical accents compared to weaker metrical accents, and this effect was predicted to be larger for errors produced in long contexts compared to short contexts. Excerpt error rates broken down by metrical accent strength were adjusted for the number of opportunities for an error to receive that metrical accent strength (arising from unequal numbers of events aligned with metrical accent strengths, shown in Figure 1). The metrical accent strength of each event corresponds to the number of Xs shown for that event in Figure 1. Each excerpt contained four events aligned with a metrical accent strength of 1, two aligned with a metrical accent strength of 2, one aligned with an accent strength of 3, and one aligned with an accent strength of 4.

**Figure 5:**
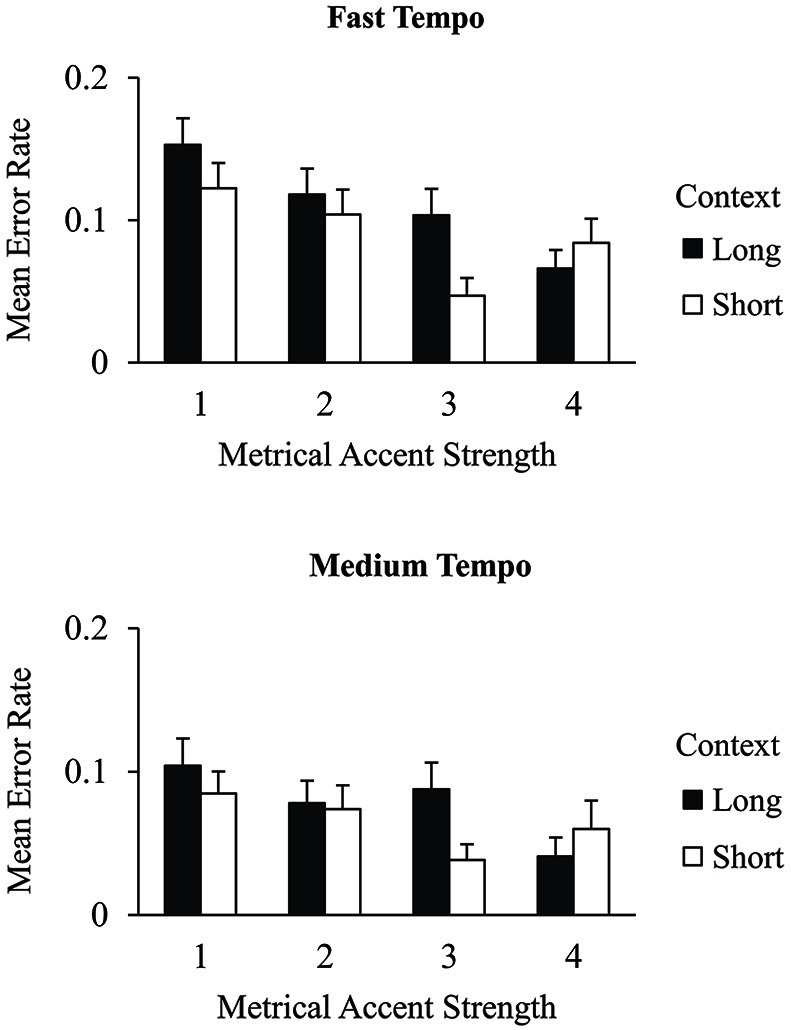
**Mean excerpt error rates for all excerpt errors by context condition and metrical accent strength at the fast tempo (top) and medium tempo (bottom)**. Error bars represent one standard error.

Figure 5 shows mean overall error rates across events within the excerpts as a function of metrical accent, context, and tempo. A 2 (context) × 2 (tempo) × 4 (metrical accent) ANOVA yielded a significant main effect of tempo, *F*_(1,25)_ = 7.50, *p* < 0.05, and a significant main effect of metrical accent, *F*_(3,75)_ = 30.23, *p* < 0.001. Overall, error rates were higher at the faster tempo (*M* = 0.10, *SE* = 0.006) than at the medium tempo (*M* = 0.07, *SE* = 0.006). Thus, the tempo manipulation reliably altered pianists’ error rates. In terms of metrical accents, error rates were lowest at positions receiving a metrical accent strength of 3 (*M* = 0.07, *SE* = 0.008) and 4 (*M* = 0.06, *SE* = 0.008), next lowest at positions receiving a metrical accent strength of 2 (*M* = 0.09, *SE* = 0.009), and highest for positions receiving a metrical accent strength of 1 (*M* = 0.12, *SE* = 0.009) (Tukey HSD = 0.040, *α* = 0.05). The main effect of context was not significant (*p* = 0.06). Critically, the context × metrical accent interaction was significant, *F*_(3,75)_ = 13.15, *p* < 0.001. Error rates for excerpts in long contexts were significantly lower for positions receiving a metrical accent strength of 4 (*M* = 0.054, *SE* = 0.009) compared with all other accents, and higher for positions receiving a metrical accent strength of 1 (*M* = 0.13, *SE* = 0.014) compared with all other accents (Tukey HSD = 0.032, *α* = 0.05). The only significant differences seen for short contexts were reduced error rates at positions receiving an accent strength of 3 (*M* = 0.043, *SE* = 0.008) compared with accent strengths of 1 (*M* = 0.10, *SE* = 0.012) and 2 (*M* = 0.089, *SE* = 0.012). There were no other significant interactions.

We further explored the differences in error rates across contexts and metrical levels using a contrast analysis (Keppel and Wickens, 2004). For each participant and context condition (based on crossing context with tempo), we derived a contrast value based on the summed products of excerpt error rates within that cell with a set of weights that linearly contrasted error rates at positions associated with each of the 4 metrical accent strengths. Negative values of this contrast indicate decreasing error probability with increasing metrical accent strength. A 2 (context) × 2 (tempo) ANOVA on the contrast measures yielded a significant main effect of context, *F*_(1, 25)_ = 6.24, *p* < 0.05, and main effect of tempo, *F*_(1,25)_ = 5.54, *p* < 0.05, but no interaction. Contrast values were generally negative, indicating a linear decrease in error probability with increasing metrical accent (errors were least likely at the most strongly accented positions), but the magnitude of contrasts was greater for long contexts (*M* = −0.11, *SE* = 0.014) than for short contexts (*M* = −0.07, *SE* = 0.012), and for fast tempo sequences (*M* = −0.11, *SE* = 0.015) than for medium tempo sequences (*M* = −0.07, *SE* = 0.011). Thus, longer contexts and slower tempi enhanced beneficial effects of metrical accent on error rates.

#### Range of planning

We tested the prediction that error types are affected by context; we predicted that the target-intruder distance for contextual excerpt errors would increase as the size of the surrounding context increased. The mean range between each contextual pitch error and the sequence position for which that pitch was intended was calculated for all contextual errors by averaging the absolute distances between the location of contextual errors and their sources within 8 events of the target event (one complete metrical cycle at the highest metrical level). A two-way ANOVA on mean ranges for excerpt errors revealed a main effect of context, *F*_(1,25)_ = 12.98, *p* < 0.005. Target-intruder distances were greater when the same excerpt was placed in a longer context (*M* = 2.1 events, *SE* = 0.07) than in a shorter context (*M* = 1.8 events, *SE* = 0.07), when possible target-intruder distances were controlled for across context length, indicating that context facilitated a greater range of planning. There was no effect of tempo or interaction with context.

To assess the possible influences of pitch repetition rates in the musical sequences on error distance measures, we analyzed the distances over which pitches recurred across the entire musical sequence and applied that criterion (*M* = 6 tones apart) to include only those errors whose distances fell within this chance estimate; this is an important control for the distributions of pitches outside the excerpt from which the errors might have arisen. The same 2 (context) × 2 (tempo) ANOVA on mean ranges of contextual errors within the pitch repetition chance estimate replicated the main effect of context, *F*_(1,25)_ = 5.50, *p* < 0.05, with greater target-intruder distances for longer contexts than shorter contexts.

#### Context-distance interactions

**Figure 6:**
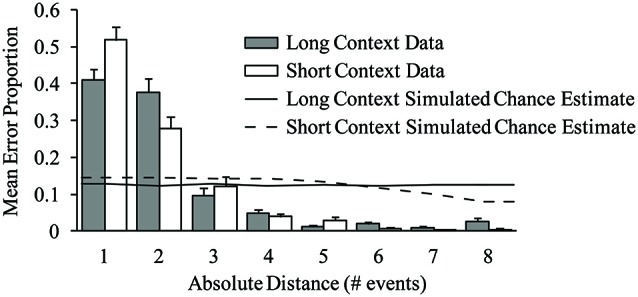
**Mean error proportions by context condition and absolute sequence distance, for contextual excerpt errors**. Error bars represent one standard error. The solid and dashed lines depict chance estimate distance proportions yielded by error simulations for the long and short context conditions, respectively.

We next tested the prediction that the pitch contents of contextual errors reflected metrically similar events more often when excerpts were placed in long contexts as opposed to short contexts. Metrically similar events should arise from sequence distances 2, 4, 6, and 8, due to the alternating strong-weak binary meter of the musical sequences used in the experiment. Contextual error proportions within each performance were computed for errors arising from distances 1 through 8, called movement gradients (Dell et al., 1997). The error proportions refer to the frequency with which each contextual error was associated with an intruder from a specific distance, and apply only to contextual errors. (This is distinct from error rates, which refer to the frequency with which an error of any sort occurs.) Mean movement gradients are shown in Figure 6 by context and distance; most errors arose from nearby distances (as predicted by the serial proximity component of the range model; Palmer and Pfordresher, 2003). An 8 (distance) × 2 (context) × 2 (tempo) ANOVA on error proportions revealed a significant main effect of distance, *F*_(7,175)_ = 139.49, *p* < 0.001, and a significant distance × context interaction, *F*_(7,175)_ = 4.78, *p* < 0.001. *Post hoc* comparisons revealed that a significantly larger proportion of errors arose from distance 1 than distance 2 in short contexts: This was not the case in long contexts. The tempo × distance interaction was also significant, *F*_(7,175)_ = 2.25, *p* < 0.05, but did not survive a Greenhouse-Geisser correction (*p* = 0.106). Importantly, and in line with model predictions, differences across contexts reflected greater accessibility of metrically similar events (even numbered positions) and lower accessibility of dissimilar events (odd numbered positions) for the long context. Differences attributable to metrical similarity across contexts were only apparent at close serial proximities, which is consistent with the range model’s prediction that metrical similarity effects are lessened at far distances due to the modulating effect of serial proximity (Palmer and Pfordresher, 2003).

To test whether longer sequence contexts affect performers’ conceptions of metrical similarity, we compared the proportions of contextual errors arising from metrically similar events (distances 2, 4, 6, and 8) across long and short contexts. On average, 44% (*SE* = 4.0%) of contextual excerpt errors in long contexts arose from metrically similar distances, whereas only 31% (*SE* = 2.7%) of excerpt errors in short contexts arose from metrically similar distances,* t*_(25)_ = 2.87, *p* < 0.005 (one-tailed). Thus, metrically similar errors (those arising from distances 2, 4, 6 and 8) were more likely to arise in long contexts than in short contexts, similar to the model’s predicted context effects.

In a second follow-up analysis, contextual error proportions were computed for errors arising from distances 1 through 4. The same 4 (distance) × 2 (context) × 2 (tempo) ANOVA on contextual error proportions revealed a significant main effect of distance, *F*_(3,75)_ = 79.62, *p* < 0.001, and a significant distance × context interaction, *F*_(3,75)_ = 3.92, *p* < 0.05. *Post hoc* comparisons revealed that a significantly larger proportion of errors arose from distance 1 than distance 2 in short contexts: This was not the case in long contexts (Tukey HSD = 0.13, *α* = 0.05), similar to the analysis of error proportions arising from distances 1 through 8. The tempo × distance interaction was not significant, *F*_(3,75)_ = 2.39, *p* = 0.08. There were no other significant effects or interactions.

Finally, we tested whether the rate at which pitches repeated in the melodic sequences differed across metrical accent strengths or context lengths. The mean distance between repeating pitches at each position in the stimulus excerpts did not correlate with the metrical accent strength at each position for long contexts, *r*_(62)_ = 0.11, *p* = 0.37, or for short contexts, *r*_(62)_ = 0.04, *p* = 0.74. In a final test of whether pitch repetitions within the musical sequences differentially influenced contextual errors in long and short contexts, we correlated the distances at which pitches repeated within each excerpt across the long and short contexts; the pitch distances were highly correlated across context lengths for both the right hand part, *r*_(62)_ = 0.90, *p* < 0.001, and the left hand part, *r*_(62)_ = 0.94, *p* < 0.001. This finding follows from the fact that the pitch contents (4 events) on either side of the excerpt were identical across long and short contexts.

### Estimating random process effects

To address whether the distance effects on error proportions shown in Figure 6 arose from a random process, we simulated performance errors by randomly sampling from the set of sequence pitches. Specifically, we simulated error events with a probability of 0.10 to match the observed error rate by randomly selecting pitches from all sequence events. We then computed the serial distance between each contextual error location and the nearest same pitch occurring in the musical sequence for errors occurring with the excerpt. One thousand error simulations were conducted for each context condition and participant to yield chance estimates that minimized the influence of noise in the output. The solid and dashed lines in Figure 6 show the mean distance proportions yielded by the error simulations for long and short context conditions, respectively. As can be seen, there is a slight tendency for contextual error frequencies to decrease with distance, and there is no evidence of metrical similarity (greater predicted proportions at even-numbered distances).

We evaluated additional factors that may influence the error patterns reported here. Previous findings (Palmer and van de Sande, 1993, 1995) indicated that pianists’ error patterns yielded hand differences (more errors produced by the left hand), diatonic relatedness (more in-key than out-of-key errors) and harmonic relatedness (more in-chord than out-of-chord errors) at rates greater than expected by chance. In the current study, 60% of all errors were produced by the left hand (greater than the chance estimate of 50%); 83% of errors yielded in-key outcomes (chance estimate = 7/12) and 49% of errors yielded in-chord outcomes (chance estimate = 3/12), similar to previous findings. Furthermore, tones produced by participants’ right hands were performed with greater intensity on average (*M* = 74.7, *SE* = 0.48) than tones produced by participants’ left hands (*M* = 58.8, *SE* = 0.46), *F*_(1,25)_ = 237.32, *p* < 0.001, similar to previous studies (Palmer and van de Sande, 1995). The patterns of meter and context effects were the same across hands. To assess pitch similarity metrics, all pitch errors were also coded in terms of the number of semitones between the pitch error and the intended pitch. Error proportions did not decrease systematically over semitone distances. Pitch error distances were also examined in terms of the nearest tone generated by the same finger movement, based on notated fingerings provided to pianists, and in terms of whether they were anticipatory (if the error source occurred ahead of the intended pitch; *M* = 42% of contextual errors) or perseveratory (if the error source occurred behind the intended pitch; *M* = 58% of contextual errors). Proportions of all pitch errors coded in terms of finger distance did not correlate significantly with the observed movement gradients in Figure 6, *r*_(6)_ = 0.41, *p* = 0.32. ANOVAs on the additional factors by context and metrical accent strength yielded no significant differences.

### Model fits

#### Range of planning

The range model was fit to individuals’ excerpt error proportions from the movement gradients separately for each context-tempo condition. Fits were carried out in two steps, following the same procedure introduced by Palmer and Pfordresher (2003). All model fits used the mean produced IOI per trial as the value for parameter *t*. The model was first fit to each individual’s movement gradients for each condition using one free parameter, *a* from Equation 1, which was allowed to vary across individuals (taking the average *a* from an initial fit to each condition for each participant, as in Palmer and Pfordresher, 2003). The metrical weight on level 2, *w*_2_ (the eighth-note level), which weights different values of *m* in Equation 2, was fit to each experimental condition, based on previous findings that this level was weighted heavily with similar pieces possessing the same 4/4 meter (Palmer and Pfordresher, 2003). The metrical weight on level 2 was permitted to vary between 0.25 and 0.99, and remaining metrical weights were set equal to ((1−*w*_2_) / (*k*−1)), where *k* represents the total number of metrical levels, as described in the introduction. Of the 26 pianists, 3 pianists produced no errors in the long-medium condition, 3 produced no errors in the short-medium condition, and 2 produced no errors in the short-fast condition; model fits could not be performed on these individual conditions. We fit only those combinations of participant and condition for which there were error data (*n* = 96).

The model provided a significant fit in 85 of the 96 individual experimental condition fits (*p* < 0.05), and the mean variance accounted for (VAF) values per individual ranged from 0.46 to 0.91 (critical VAF = 0.50); fits by individual were significant for 23 out of 26 individuals (*p* < 0.05; *M* individual VAF = 0.75). The model’s parameter *a*, which is thought to capture individual working memory constraints, was correlated with the behavioral metric for mean range based on error distances. A significant positive correlation was found between individuals’ *a* parameter values (range = 0.77–0.89) and their mean range of planning (the mean distance between each contextual error and its source), *r*_(24)_ = 0.46, *p* < 0.05. Thus, participants with higher *a* values had greater mean ranges of planning, similar to previous findings (Palmer and Schendel, 2002; Palmer and Pfordresher, 2003).

#### Metrical component

**Figure 7:**
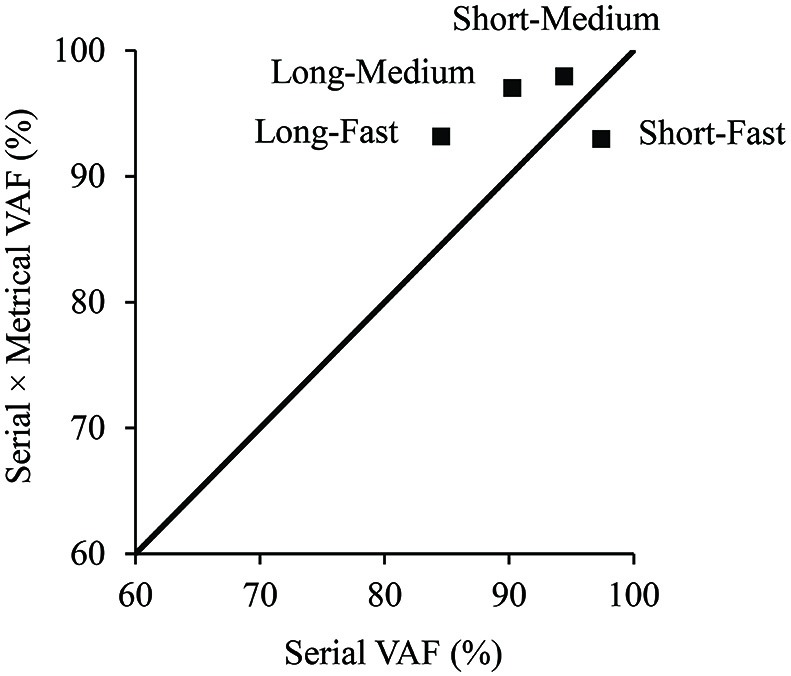
**Percent variance accounted for in the mean excerpt error proportions for each tempo-context condition by the serial model (x-axis), compared with the serial × metrical model (y-axis)**.

We tested whether the model’s metrical similarity component was necessary to explain the excerpt errors by first comparing two different versions of the model: one containing the metrical component (*S* × *M*) and the other without the metrical component (*S*). Both the meter-free model (*S*) and serial × metrical (*S* × *M*) model were first fit to the mean data for each tempo-context condition with *a* varying, and subsequent fits used *a* values averaged across conditions. All metrical levels were weighted equally (*w_j_* = 0.25) for fits of the serial × metrical (*S* × *M*) model so that the number of free parameters was identical to the meter-free (*S*) model. Figure 7 shows the VAF for the mean movement gradients fit by the model that contained only the serial component (*S*) and one containing the metrical component in addition (*S* × *M*). The diagonal line in Figure 7 indicates values for which the models with and without the metrical component would explain equivalent variance. Three of four conditions fall above the diagonal line, indicating value added by the metrical component in those conditions. Thus, inclusion of the model’s metrical component was able to explain more variance in three of the four conditions: both of the long context conditions (the two conditions expected to enhance metrical similarity) and the short-medium condition. The lower half of Table 1 shows the mean VAF for fits of the (*S*) and (*S* × *M*) models to individual subject data for each of the 4 tempo-context conditions. The (*S*) model provided a significant fit in 58 of the 96 individual experimental condition fits, and the (*S* × *M*) model provided a significant fit in 72 of 96 individual fits (*p* < 0.05). Thus, inclusion of the metrical component was able to explain more variance for the majority of individual data.

**Table 1:** **Goodness-of-fit of the serial (S) and serial × metrical (S × M) models to mean error patterns, and mean model fits to individual data**.

			*S*		*S* × *M* (*w_j_* fixed)		*S* × *M* (*w_j_* varied)	
	Condition	*N*	VAF (%)	AIC	VAF (%)	AIC	VAF (%)	AIC
Mean data	Long-Medium	23	84.0	−26.0	97.0	−34.2	98.4	**−36.7**
	Long-Fast	26	73.7	−22.1	93.2	−27.8	99.3	**−41.3**
	Short-Medium	23	91.9	−29.0	98.0	**−35.9**	98.0	−33.9
	Short-Fast	24	99.4	**−32.5**	93.0	−25.5	93.0	−23.5
Mean of individual fits	Long-Medium	23	52.3	−14.3	60.2	−14.8	75.0	**−17.1**
	Long-Fast	26	52.0	−16.1	63.5	−16.7	79.9	**−20.0**
	Short-Medium	23	48.4	−10.9	54.5	−11.5	69.8	**−13.7**
	Short-Fast	24	79.8	**−22.2**	77.0	−19.5	77.6	−18.3

*Note. Bold values indicate the best-fitting model for each tempo-context condition*.

Finally, we tested whether the model’s metrical parameter, *w*_2_, provided additional explanatory value for the behavioral measures. When the metrical parameter was permitted to vary in fits of the serial × metrical (*S* × *M*) model, the VAF was improved for all four conditions over the serial (*S*) model and serial × metrical (*S* × *M*) model fits, shown in the upper half of Table 1. The Akaike information criterion (AIC; Akaike, 1973), a measure of model complexity that takes into account the number of model free parameters, was computed for the fits shown in Table 1 (a lower value indicates a better fit). As shown in Table 1 (column 3), the serial × metrical (*S* × *M*) model with the additional free parameter *w*_2_ provided a better fit for the 2 long context conditions, but not the 2 short context conditions. Thus, the *S* × *M* model’s additional metrical weight parameter, derived from fits of the model to the data, improved the long context fits above and beyond the introduction of the model’s metrical similarity component (column 2 of Table 1). The bold values in Table 1 indicate the best-fitting model for each tempo-context condition. The (*S* × *M*) model with *w_j_* fixed at 0.25 provided a significant fit in 72 of the 96 individual experimental condition fits, and the (*S* × *M*) model when *w*_2_ was allowed to vary provided a significant fit in 85 of 96 individuals fits (*p* < 0.05). Thus, inclusion of the metrical weight on level 2 (an intermediate metrical level) was able to explain more variance for the majority of individual data.

A 2 (tempo) × 2 (context) ANOVA on individuals’ fitted metrical weights (*w*_2_ values) yielded a significant main effect of tempo, *F*_(1,25)_ = 6.49, *p* < 0.05. Excerpt errors from medium tempo conditions yielded model fits with larger metrical weights (*M w*_2_ = 0.48, *SE* = 0.036) than excerpt errors from fast tempo conditions (*M w*_2_ = 0.40, *SE* = 0.033). Fits of the model to the data from long context conditions tended to result in larger metrical weights (*M w*_2_ = 0.48, *SE* = 0.037) than fits to data from short context conditions (*M w*_2_ = 0.40, *SE* = 0.032), although the main effect of context on metrical weights was not significant (*p* = 0.06). The interaction between tempo and context variables was not significant. Thus, the metrical weighting parameter indicated greater emphasis on level 2 of the metrical hierarchy when excerpts were performed at a slower tempo and in a longer context.

## Discussion

Musicians’ production errors, elicited in performances of musical excerpts that were surrounded by long and short contexts, indicated three facilitative effects of a longer musical context. First, serial ordering errors arose between sequence events that spanned greater distances when excerpts were placed in longer contexts than in shorter contexts. This finding suggests that performers employed greater ranges of planning for events in long contexts, consistent with the range model’s (Palmer and Pfordresher, 2003; Pfordresher et al., 2007) prediction that memory activation of current events arises from their contextual relationships. Second, more serial ordering errors arose from metrically similar sequential positions for events placed in longer contexts than in shorter contexts. The enhanced metrical similarity effect may reflect the influence of higher-level hierarchical relationships that are defined over longer sequence distances, corroborated by evidence for metrical hierarchies in musicians’ keystroke intensities for both correct and erroneous pitches. Third, longer sequence contexts increased the tendency for pitch events aligned with relatively stronger metrical accents to be performed with greater accuracy. Each of these findings follows from the fact that larger metrical periodicities are defined over greater sequence distances in longer contexts than in shorter contexts, and the salience of events from greater distances enhances performers’ ability to plan larger increments during performance.

These findings underscore the role of contextual information in the retrieval of musical sequences. Contextually defined hierarchies of musical features may provide performers with a means of quickly and efficiently retrieving information from memory, consistent with theories of expert performance memory (Ericsson and Kintsch, 1995; Chaffin and Imreh, 1997). Our findings support the proposal that metrical accent, one musical feature that is defined by the sequence context, provides such a framework for planning (Palmer and Pfordresher, 2003). Longer sequence contexts contain information to define hierarchical relationships that span larger time periods; this was evidenced by the larger proportions of metrically similar errors in long than in short sequence contexts. Thus, longer sequence contexts may enhance the overall magnitude of metrical hierarchies more than shorter contexts, a notion that is supported by evidence that metrical representations take time to become instantiated during listening (Longuet-Higgins and Lee, 1982; Drake and Botte, 1993). The strength of neural oscillations underlying metrical hierarchies may also therefore depend on contextual information that reinforces a metrical pattern. Fewer repetitions of higher-level metrical periodicities in short contexts during music perception may lead listeners to focus attention at lower periodicities, whereas longer contexts provide more stimulus beats for attentional pulses to shift to higher metrical levels (Large and Jones, 1999; Large and Palmer, 2002). The greater salience of higher-order metrical accents in the longer sequences, also evidenced in the increased intensity with which performers produced those events, is likely to have contributed to larger planning ranges.

### Comparison with other approaches

The range model’s approach to the role of contextual information in memory retrieval can be contrasted with the approach taken by temporal-contextual distinctiveness theories of serial recall (e.g., Brown et al., 2000, 2007) which predict that memorability of a sequence event increases as temporal separation from neighboring contextual events increases. Brown et al.’s (2007) scale-independent memory, perception, and learning (SIMPLE) model, for example, proposes that event retrieval is constrained by how easily items can be temporally discriminated in terms of their position, similar to the identification of stimuli in terms of position along dimensions such as weight, line length, or loudness. In SIMPLE, an item is best retrieved when it is temporally isolated from contextual events: Retrieval is most accurate when little (or no) contextual information is present. Thus, SIMPLE can be described as taking the view that contextual information is detrimental to retrieval of the current event. The range model proposes the opposite: The larger the sequence context, the greater the likelihood that the current event tied to that context will be retrieved.

The oscillator-based associate recall (OSCAR) model of Brown et al. (2000; Vousden et al., 2000) also predicts a beneficial effect of temporal isolation on recall. OSCAR proposes that sinusoidal oscillators encode contextual information in sequences. Retrieval of a sequence event from memory is achieved by reinstating the oscillator pattern with which the event is associated. Sequence events that are more temporally distinctive are associated with different combinations of oscillator activations than events that are temporally close together, and are as a result retrieved more easily. The findings presented here qualify theories of temporal distinctiveness like SIMPLE and OSCAR by demonstrating that increases in the number of contextual events surrounding a current event can facilitate event preparation, as opposed to hindering it.

The range model differs from other models in its claim that contextual information is not only accessible during production, but also makes the current event *more* accessible than it otherwise would have been if there were *less* (or no) sequential context. Most models of memory retrieval have neglected to incorporate hierarchical structure in theoretical frameworks. Research on effects of hierarchical structures on recall has led to the acknowledgment that short-term memory retrieval processes must *somehow* interact with information stored in long-term memory (Atkinson and Shiffrin, 1968; Crowder, 1976; Henson, 1998; Burgess and Hitch, 2005). The gap between short-term retrieval processes and hierarchical facilitation is theoretically bridged with a “unitary” view of short- and long-term memory, which proposes that short-term memory representations are simply the contents of long-term memory in an activated state (Cowan, 1995, 2001). Another approach is Botvinick and Plaut (2006) recurrent network account of serial recall, which relies on an activation-based mechanism of recurrent connectivity that adjusts patterns of unit activation for domain-specific knowledge. Connectionist models typically provide mechanisms for both bottom-up and top-down influences on perception, in which context can constrain which information is activated in memory (Thomas and McClelland, 2008). The metrical framework proposed here similarly reflects producers’ learned knowledge of events’ accent patterns, which may modulate the spread of activation within distributed neural networks during sequence production.

The current study tested range model predictions with musical pieces that contained isochronous events in order to maintain equivalent rates of musical events occurring at different metrical positions. Because strong and weak metrical accents alternate strictly in binary meters across all sequence positions, metrical similarity is not confounded with primacy or recency effects. Much of Western tonal music contains varying tone durations, which can influence chunking processes, in which retrieval of musical segments is an all-or-none process cued by longer durations occurring at chunk boundaries (Ericsson and Kintsch, 1995; Chaffin and Logan, 2006). However, most errors during performance of rhythmically varying music, like isochronous music, involve single pitches and not larger units or chunks (Palmer and van de Sande, 1993; Drake and Palmer, 2000), a finding corroborated by the current study, which supports the idea that performers possess incremental access to contextual sequence events during production. Future studies may test extensions of range model predictions to rhythmically varying music, by incorporating a duration-based similarity metric. For example, two tones could share the same metrical accent strength, enhancing hierarchically-based similarity, but differ in terms of their durations, decreasing their overall similarity.

The musical pieces used in the current study were characterized by binary metrical structures that contained a strict alternation of strong and weak beats. An analysis of a large corpus of musical styles has shown that the frequency with which pitches occur in different metrical locations can differ for binary and ternary meters and can differ across musical styles and genres (Palmer and Pfordresher, 2003). Furthermore, the beat level of a metrical hierarchy may also consist of combinations of binary and ternary accents, as in complex meters (London, 1995). It remains to be tested whether different musical meters and styles lead to different distributions of contextual errors. The musical pieces used in the current study presented short (8-event) excerpts in relatively short musical contexts; future studies should also employ contexts of greater length, in order to examine further context effects on planning. Larger contexts may heighten facilitative effects on planning as the range model predicts, or perhaps those contexts would reach a threshold at which contextual relationships could no longer be integrated in an updated memory representation. The relatively brief contexts used in the current study (25 non-excerpt events for the large context and 9 non-excerpt events for the short context) limited the range of observable serial ordering errors for some positions in the small context excerpt within an 8-event hierarchical metrical cycle. The size of the contexts and the windows used to identify contextual error sources influences the numbers of strongly and weakly accented contextual event positions, which were kept constant in the current study; nonetheless, analyses that limited the size of the window to exclude events outside chance estimates yielded the same findings as were observed with a larger (8-event) window. Furthermore, we simulated the error types that would result from simple variations in the size of the event window; these error patterns differed completely from the experimentally observed errors.

Metrical hierarchies may modulate influences on memory of other musical dimensions such as tonality, harmony, and timbre, which also yield similarity-based confusion errors in comprehension and performance (Crowder and Pitt, 1992; Palmer and van de Sande, 1993). For example, metrical accents can interact with pitch accents in memory for musical sequences (Jones, 1987), and perceptual experiments suggest that listeners combine multiple pitch and temporal features from which they infer meter (Hannon et al., 2004). Thus, production errors that incorporate other musical dimensions may yield similar contextual predictions to the ones tested here. Pianists were instructed to use the same notated fingerings for excerpts placed in short and long contexts. Thus, although some fingerings are easier than others (and may have contributed to error likelihoods), it is unlikely that fingerings accounted for the context effects reported here.

Predictions of the range model can be applied to the production of other complex sequences, such as typing and speech, whose elements hold hierarchical relationships defined within each context (cf. Crump and Logan, 2010). The core assumptions of the range model for memory retrieval are implemented with a parameter space constrained by similarity-based interference processes that arise within the constraints of working memory. The range model has been applied to the production of nonsense syllables (Palmer and Schendel, 2002); metrical patterns arising from varying degrees of syllable prominence (Liberman and Prince, 1977) can be captured by hierarchical periodicities in the range model’s metrical component. The current findings suggest that longer contexts in spoken language may lead to greater stress placed on linguistic units that are aligned with greater hierarchical periodicities, as metrical relationships among elements would become more firmly established with increased context.

## Conclusion

Sometimes more is better: Increased metrical associations in longer sequential contexts facilitate performers’ retrieval of individual sequence events. Incrementally planned representations are popular among memory retrieval models during sequence production; the range model (Palmer and Pfordresher, 2003; Pfordresher et al., 2007) makes unique assumptions about similarity-based relationships among metrically regular sequence events. Advantages for sequence planning incurred from longer contexts, therefore, temper the negative outcomes of list length effects on retrieval processes: Increased contextual information is not always detrimental to memory retrieval.

## Conflict of interest statement

The authors declare that the research was conducted in the absence of any commercial or financial relationships that could be construed as a potential conflict of interest.
